# Genetic and Social Determinants of Post-Traumatic Stress Disorder: What Is Ready for Clinical Practice? A Critical Integrative Review

**DOI:** 10.3390/jcm15218468

**Published:** 2026-10-30

**Authors:** Ghazi I. Al Jowf

**Affiliations:** Department of Public Health, College of Applied Medical Sciences, King Faisal University, Al-Ahsa 31982, Saudi Arabia; galjowf@kfu.edu.sa

**Keywords:** post-traumatic stress disorder, PTSD, genetic determinants, social determinants, epigenetics, biomarkers, personalized diagnostics, precision psychiatry, polygenic risk, machine learning

## Abstract

Post-traumatic stress disorder (PTSD) is diagnosed clinically, yet similarly exposed individuals follow markedly different courses, and clinicians increasingly encounter claims that genetic, epigenetic, or social information can personalize assessment. This critical integrative review asks which of these factors is ready to inform clinical decisions. PubMed/MEDLINE, Scopus, and Web of Science were searched from inception to August 2026, prioritizing meta-analyses, large multi-cohort genomic and epigenomic studies, prospective cohorts, and externally validated prediction models. Genome-wide studies now implicate 95 risk loci and confirm a highly polygenic architecture, but no genetic, epigenetic, neuroendocrine, or inflammatory marker functions as a stand-alone diagnostic test. In contrast, trauma dose, childhood adversity, socioeconomic instability, social support, displacement, and occupational exposure are clinically actionable now for triage and follow-up planning, although they are not diagnostic evidence. This review proposes a four-tier bio-social calibration framework that separates exposure context, the validated clinical phenotype, research-grade biological adjuncts, and longitudinal monitoring, and that rejects adjusting diagnostic criteria by genotype or social profile. In current practice, structured trauma and social assessment with measurement-based follow-up is the evidence-supported approach; biological markers should remain research tools until they demonstrate incremental value, cross-ancestry calibration, and external validation.

## 1. Introduction

Post-traumatic stress disorder (PTSD), rather than “post-traumatic stress syndrome,” is the established diagnostic term for a trauma- and stressor-related disorder characterized by persistent intrusion, avoidance, negative alterations in cognition and mood, and alterations in arousal/reactivity after a qualifying traumatic exposure [1,2]. Symptoms must persist beyond the immediate post-trauma period and produce clinically important distress or impairment. Contemporary accounts emphasize that PTSD is not a unitary fear disorder: dysregulated threat learning, contextual memory, salience processing, stress-endocrine signaling, immune biology, sleep, and cognitive control can converge on similar clinical phenotypes [1,2,3]. Traumatic-event exposure is common worldwide, whereas PTSD develops in only a subset of exposed individuals and varies by trauma type, sex, prior adversity, social context, and population. Cross-national surveys and national epidemiologic studies demonstrate substantial heterogeneity in both exposure and conditional risk, while trajectory studies show that chronic, delayed, recovering, and resilient courses can coexist after superficially similar events [4,5,6,7]. This heterogeneity creates a diagnostic problem: the presence of trauma alone is neither necessary evidence of current PTSD nor sufficient to predict its later emergence [4,7].

Diagnosis therefore remains clinical and is based on the criteria of the Diagnostic and Statistical Manual of Mental Disorders, Fifth Edition (DSM-5). The Clinician-Administered PTSD Scale for DSM-5 (CAPS-5) provides a structured clinician assessment of symptom presence and severity, while the PTSD Checklist for DSM-5 (PCL-5) is a validated self-report instrument useful for screening and monitoring [8,9]. These tools operationalize the phenotype but do not directly encode inherited susceptibility, biological embedding of adversity, social resources, cultural expression, or future course. A checklist score can also be influenced by literacy, stigma, somatic idioms of distress, disclosure, comorbidity, and the timing of assessment. The limitation is not that DSM-based criteria are biologically “wrong”; it is that a categorical syndrome definition answers a different question from individualized risk estimation.

Pre-trauma and early post-trauma prediction studies illustrate the distinction. Models based on background characteristics can identify gradients of risk, and multinational early-survivor data improve prediction when acute symptoms and exposure features are added, but performance varies across settings and does not justify deterministic labeling of an individual [10,11]. This is especially important when risk information could affect military duties, insurance, migration processes, employment, or access to scarce services. A useful personalized diagnostic system must improve decisions without converting probabilistic correlates into quasi-diagnostic labels [8,9,10,11].

The rationale for a dual-determinant approach is therefore mechanistic and clinical. Genetic variation can shape stress responsivity, learning, neuroplasticity, and sensitivity to environmental inputs, yet genetic effects are distributed across many variants and are expressed within developmental and social environments. Conversely, social determinants can alter trauma exposure, chronic stress burden, recovery resources, treatment access, and symptom communication, but they do not explain all between-person variation. Treating either domain as sufficient obscures interaction, mediation, selection, and biological embedding [1,2,3,7].

This review asks a narrower and more clinically useful question than whether a factor is “associated with PTSD”: what evidence would be required for that factor to improve diagnosis, triage, or longitudinal reassessment? This review synthesizes stress-response, monoaminergic and neuroplasticity candidate-gene literature; large-scale genome-wide evidence; epigenetic findings; trauma, socioeconomic, cultural, developmental, occupational, and community determinants; and emerging biomarker and machine-learning approaches. The review contributes a bio-social calibration framework that distinguishes etiologic association from diagnostic readiness and stages information according to intended clinical use, transportability, and fairness.

## 2. Methods

### 2.1. Search Strategy

This is a targeted critical integrative review, not a registered systematic review. PubMed/MEDLINE, Scopus, and Web of Science were searched from database inception to 31 August 2026; Google Scholar was used to identify additional relevant papers and citation links, and the reference lists of key reviews, meta-analyses, and major primary studies were screened backward. Searches combined three concept groups with AND, with related terms within each group combined with OR: (1) PTSD or post-traumatic stress disorder; (2) genetic, epigenetic, biological, or social determinant terms, including genetics, genome-wide association studies (GWAS) candidate genes, *FKBP5 (FK506-binding protein 5)*, *CRHR1 (corticotropin-releasing hormone receptor 1)*, *NR3C1 (glucocorticoid receptor gene)*, *5-HTTLPR (serotonin-transporter-linked polymorphic region)*, *COMT (catechol-O-methyltransferase)*, *BDNF (brain-derived neurotrophic factor)*, polygenic risk, and polygenic scores; epigenetics, methylation, epigenome-wide association studies (EWAS), hypothalamic–pituitary–adrenal (HPA) axis, cortisol, inflammation, biomarkers, and multi-omics; and social determinants, childhood adversity, socioeconomic status or instability, social support, culture, refugees, displacement, and occupational trauma; and (3) diagnosis, diagnostic, prediction, personalized diagnostics, precision psychiatry, or machine learning. PubMed searches combined Medical Subject Headings (MeSH) and free-text terms in title and abstract fields, Scopus searches used title, abstract, and keyword fields, and Web of Science searches used topic fields. Searches were limited to English-language studies in humans, with no lower publication-year limit.

### 2.2. Evidence Selection

Titles and abstracts were screened for relevance to the genetic, epigenetic, neuroendocrine, inflammatory, social, diagnostic, or predictive aspects of PTSD, and potentially relevant records were assessed in full text. Peer-reviewed human evidence with sufficient methodological detail was retained when it directly informed PTSD susceptibility, biological embedding, social determinants, diagnostic assessment, risk stratification, prediction, clinical utility, or translational readiness. Priority was given to systematic reviews and meta-analyses, large multi-cohort GWAS and EWAS, prospective cohort studies, and externally validated prediction models; influential primary studies were also retained when they provided mechanistic, clinical, diagnostic, or translational evidence not adequately represented in higher-level reviews. Records were excluded if PTSD was not a primary focus and PTSD-specific findings could not be separated, if they reported animal-only data, if they were editorials, commentaries, or opinion pieces without primary or synthesized evidence, if they duplicated other reports, or if they were small candidate-gene studies superseded by larger replicated evidence. Screening and selection were performed by the author. Influential primary studies were defined as first reports of a mechanism or method on which later work built, or as the largest or most recent study within their domain. Consistent with the narrative design, screening was iterative and record counts at each stage were not logged; 68 sources were included in the final synthesis.

### 2.3. Synthesis and Appraisal

Evidence was synthesized narratively and organized by genetic determinants, social determinants, convergence mechanisms, and diagnostic readiness, with particular attention to clinical actionability, transportability, fairness, and external validation. Because the objective was evidence integration rather than pooled effect estimation, no meta-analysis, Preferred Reporting Items for Systematic Reviews and Meta-Analyses (PRISMA) flow diagram, or formal risk-of-bias tool was applied. Instead, each study was appraised according to its design, sample size, replication, external validation, confounding, tissue specificity, ancestry portability, and clinical relevance. Where the summary figures use ordinal 0–3 ratings, these are the author’s interpretive, single-rater judgments. Each rating weighed the study design, sample size, replication, external validation, and clinical applicability of the evidence summarized in Section 3, Section 4, Section 5 and Section 6 and Table 1 and Table 2, using the anchor definitions given in each figure legend. The ratings organize the evidence and are not pooled effect sizes.

## 3. Genetic Determinants of PTSD

### 3.1. Heritability and Polygenic Architecture

Family and twin observations support an inherited contribution to variation in post-traumatic symptoms, but they do not imply genetic determinism. A classical twin study supports a heritable spectrum spanning vulnerability and resilience, while genome-wide studies demonstrate measurable single-nucleotide polymorphism (SNP)-based heritability and genetic correlation with other psychiatric phenotypes [12,13]. The important diagnostic implication is that inherited liability is distributed rather than binary. No single variant divides trauma-exposed people into “PTSD-prone” and “PTSD-resistant” categories, and estimates derived from one ascertainment strategy or ancestry cannot be assumed to transport unchanged to another population.

### 3.2. HPA-Axis and Stress-Response Genes

*FKBP5* is the clearest example of why genetic effects should be interpreted within exposure history. Initial work linked *FKBP5* polymorphisms with childhood abuse in relation to adult PTSD symptoms, subsequent studies reported *FKBP5*-by-childhood-adversity interaction, and mechanistic work connected risk-allele status and childhood trauma with allele-specific demethylation and glucocorticoid-response changes [14,15,16]. These findings are biologically coherent because *FKBP5* regulates glucocorticoid receptor sensitivity, but they are not a clinical genotype test: effect sizes depend on exposure definition, ancestry, age, tissue, phenotype, and study design.

The broader hypothalamic–pituitary–adrenal (HPA) axis includes *CRHR1*, *NR3C1*, and regulatory networks affecting corticotropin and glucocorticoid signaling. Reviews of gene–environment vulnerability emphasize that altered HPA feedback can emerge from both inherited variation and trauma-related regulation, making causal direction difficult to infer from cross-sectional data [17]. Pituitary adenylate cyclase-activating polypeptide (PACAP) and its type 1 receptor (PAC1) signaling provides another stress-response example; an influential study associated PTSD with PACAP and its receptor, with sex-linked patterns that stimulated mechanistic research [18]. Yet even biologically plausible candidate pathways require replication at genome-wide scale before they should influence diagnostic classification.

### 3.3. Monoaminergic and Neuroplasticity Candidates

Candidate studies have also focused on serotonergic, catecholaminergic, dopaminergic, and neurotrophic pathways. The *5-HTTLPR* literature illustrates the replication problem: meta-analytic evidence has examined an association with PTSD, but the signal is not sufficiently stable or specific to function as a diagnostic marker [19]. *COMT* Val158Met, which influences catecholamine metabolism and therefore intersects with prefrontal dopamine biology, has likewise not been supported; meta-analysis found no overall association with PTSD [20]. *BDNF* Val66Met has been reported as a possible risk factor in case–control and meta-analytic work, plausibly linking neuroplasticity, memory consolidation, and extinction learning, but the evidence remains probabilistic and population-dependent [21]. These examples caution against pathway plausibility being mistaken for clinical validity. Several factors explain why candidate-gene studies fell short: small samples, a low prior probability that any single variant carries a meaningful effect in a highly polygenic disorder, flexible definitions of trauma exposure and outcome, and selective publication of positive results. Candidate genotypes therefore have no current clinical validity for PTSD diagnosis or risk prediction.

### 3.4. From Candidate Genes to Genome-Wide Discovery

Genome-wide association studies (GWAS) changed the evidentiary standard by testing common variation without selecting a single favored pathway. Early GWAS reported loci including RORA (retinoid-related orphan receptor alpha) and other susceptibility signals, but sample sizes were modest and replication was difficult [22,23]. Sex-specific signals and associations in women were also reported, including a long intergenic non-coding RNA locus [24]. Studies in Iraq–Afghanistan veterans and U.S. Marines broadened cohorts and ancestry representation, while military samples provided more structured characterization of trauma exposure [25,26]. Two U.S. Army cohorts further demonstrated that phenotype definition and cohort composition materially influence locus discovery [27].

As consortia expanded, the architecture became clearer. International meta-analysis identified sex- and ancestry-specific loci, large veteran datasets increased power for re-experiencing phenotypes, and Million Veteran Program analyses resolved additional loci across PTSD and symptom subdomains [28,29,30]. Integrating quantitative symptoms and trauma exposure can increase discovery power, but it also changes the phenotype being modeled and therefore the interpretation of resulting scores [31]. The 2024 multi-ancestry GWAS identifying 95 risk loci represents a major advance in statistical discovery and neurobiological mapping [32]. It does not, however, convert GWAS findings into a diagnostic assay: genetic discovery, risk prediction, and clinical utility are separate validation stages.

Across this progression, the most defensible conclusion is polygenicity with heterogeneity, pleiotropy, and context dependence. Reviews moving from gene discovery to disease biology emphasize that genetic findings can illuminate pathways and shared liability even when individual-level prediction remains modest [33]. For personalized diagnostics, the priority should therefore shift from asking whether a “PTSD gene” exists to testing whether aggregate genetic information adds reproducible incremental value beyond trauma history, symptoms, and readily available clinical variables.

### 3.5. Epigenetic Modification and Biological Embedding

Epigenetic studies provide a plausible bridge between genotype and lived environment because DNA methylation and related regulatory marks can vary with developmental exposures, smoking, immune state, cell composition, medication, and disease processes. Early work reported PTSD-associated epigenetic and immune profiles, supporting the hypothesis that trauma can become biologically embedded [34]. Intergenerational research on Holocaust exposure and *FKBP5* methylation generated particular interest, but such designs cannot by themselves separate inherited effects, parental exposures, family environment, and other cross-generational pathways [35].

Larger epigenome-wide association studies (EWAS) have improved reproducibility. A meta-analysis across military and civilian cohorts identified AHRR (aryl hydrocarbon receptor repressor) methylation changes, but *AHRR* is strongly influenced by smoking and illustrates the need for careful confounder control [36]. Longitudinal military cohorts are methodologically stronger because methylation can be measured before and after deployment, yet the number of repeated samples and tissue constraints still limit causal interpretation [37]. More recent meta-analyses implicate immune and oxidative-stress genes and, across 23 military and civilian cohorts, report additional PTSD-associated methylation sites [38,39]. The direction of travel is positive, with larger samples, harmonization, and longitudinal designs, but blood methylation remains an imperfect proxy for brain regulation and is not currently a stand-alone clinical diagnostic test.

In practical terms, genetic and epigenetic markers are best viewed today as research-level stratifiers. A marker becomes diagnostically useful only when its analytical validity, reproducibility, incremental discrimination, calibration, ancestry portability, and effect on patient outcomes are established for defined clinical use. Association with case status is only the first of these steps.

Polygenic risk scores (PRS) deserve particular caution. A PTSD PRS aggregates many alleles weighted by discovery-sample effects, but the resulting number is conditional on phenotype definition, ancestry composition, genotyping and imputation quality, linkage-disequilibrium structure, and the prevalence and distribution of trauma in the target population. A score can therefore rank genetic liability without providing a clinically useful probability of current PTSD. Moreover, PTSD is exposure-dependent: genetic liability cannot be interpreted independently of whether, when, and how trauma occurred. Sex-stratified and ancestry-specific GWAS findings further imply that a universally calibrated threshold is not currently defensible. The appropriate translational experiment is not to ask whether a PRS is statistically significant, but whether it improves calibration and decision-making beyond standardized symptoms, trauma history, and social context in an independently recruited population. This distinction is essential for clinical translation.

## 4. Social Determinants of PTSD

### 4.1. Trauma Exposure: Type, Severity, Chronicity, and Timing

Social determinants begin with the structure of exposure itself. Meta-analyses show that event characteristics and post-event conditions often predict PTSD more strongly than many pre-trauma demographic variables [40,41]. Intentional interpersonal violence, perceived life threats, repeated exposure, injury, loss, and subsequent stress can produce different risk profiles from isolated non-intentional events. In children and adolescents, developmental stage, family functioning, parental response, and ongoing adversity further alter risk [42]. An umbrella review confirms that PTSD risk is distributed across pre-trauma, peri-trauma, and post-trauma domains rather than being reducible to a single exposure measure [43]. A personalized diagnostic history should therefore record trauma “dose” and context without assuming that more severe exposure necessarily maps linearly to symptom severity in every individual.

### 4.2. Socioeconomic Position, Instability, and Access to Care

Socioeconomic disadvantages can operate before, during, and after trauma. Lower socioeconomic status was a consistent, if modest, pre-trauma risk factor in early meta-analytic work [40], and genetically informed analyses suggest that economic status partly mediates the inverse association between educational attainment and PTSD [44]. Poverty and unstable housing may increase exposure to violence and disaster vulnerability, reduce control over recovery conditions, constrain transport and time for care, and increase chronic financial stress [45]. These pathways can confound simple associations between socioeconomic status and PTSD: lower socioeconomic position may be both a cause of greater exposure and a consequence of trauma-related disability. The diagnostic implication is not to treat poverty as a symptom, but to recognize it as a modifier of pretest risk, persistence, missed appointments, treatment feasibility, and the meaning of functional impairment.

### 4.3. Social Support and Interpersonal Context

Post-trauma social support is one of the most consistently observed protective correlates. Longitudinal meta-analysis supports an association between greater social support and lower later PTSD symptoms, although bidirectionality remains plausible because worsening symptoms can erode relationships [46]. Network size and perceived quality are also not interchangeable; evidence suggests that the quality of support can matter more than simply counting social contacts [47]. Clinically, asking “who is available?” is therefore less informative than assessing whether support is trusted, practical, non-stigmatizing, and sustainable.

### 4.4. Culture, Stigma, and Differential Pathways to Diagnosis

Race and ethnicity are not biological proxies for PTSD risk; they index heterogeneous histories, exposures, structural conditions, and cultural contexts. U.S. studies show group differences in traumatic-event exposure, PTSD development, and treatment seeking, and additional work demonstrates heterogeneity in prevalence and correlates across racial and ethnic minority groups [48,49]. These differences can arise from exposure distributions, discrimination, socioeconomic conditions, service access, language, trust, and measurement performance rather than innate group liability.

Cross-cultural research also challenges the assumption that the same symptom wording has identical meaning across settings. Cultural idioms, somatic expression, spiritual interpretations, family expectations, and stigma can shape disclosure and help-seeking [50]. Standardized criteria remain important precisely because they limit arbitrary reinterpretation, but administration may require validated translations, culturally competent interviewing, and attention to locally meaningful expressions of distress. Personalized diagnostics should improve measurement equivalence; it should not create different diagnostic thresholds for socially defined groups without empirical validation.

### 4.5. Childhood Adversity and Developmental Vulnerability

Childhood adversity is a particularly important convergence point because it changes both subsequent exposure probability and biological stress regulation. World Mental Health Survey analyses support stress sensitization: prior childhood adversity can increase vulnerability to PTSD following later trauma [51]. A broad meta-analysis of adverse childhood experiences also demonstrates cumulative associations with multiple later health outcomes, underscoring that adversity is transdiagnostic rather than PTSD-specific [52]. For diagnosis, childhood adversity therefore supplies context and may increase clinical vigilance, but it cannot establish PTSD in the absence of the required trauma-linked symptom phenotype.

### 4.6. Occupational and Community-Level Exposure

Some occupations produce recurrent exposure to injury, death, threat, and morally difficult events. Rescue workers show substantial PTSD burden across studies, with prevalence varying by occupation, disaster characteristics, and study methods [53]. Military personnel, emergency clinicians, firefighters, police, and other first responders may also normalize distress or fear occupational consequences of disclosure, creating under-recognition even when screening is available. Repeated occupational exposure additionally complicates selection of a single “index trauma,” which can affect structured diagnostic interviewing.

At community level, mass conflict, forced displacement, violence, and disasters combine acute traumatic events with prolonged social disruption. Meta-analysis in conflict-affected and displaced populations links cumulative potentially traumatic experiences with adverse mental-health outcomes [54]. Refugees and asylum seekers show high and heterogeneous mental-health burden across host settings, but estimates are shaped by sampling, legal status, premigration trauma, postmigration adversity, and access to services [55]. Disaster research similarly emphasizes that exposure severity interacts with bereavement, displacement, economic loss, community resources, and recovery trajectories [45,56]. These are not peripheral “social variables”; they can determine whether risk persists after the precipitating event has ended.

Sex differences add another layer of heterogeneity. A quantitative review found that trauma types and PTSD risk differ between women and men, but observed differences cannot be reduced to sex-linked biology because exposure patterns and social context also differ [57]. This is a recurring theme across social determinants: variables such as sex, race/ethnicity, occupation, and income often bundle multiple causal mechanisms. High-quality personalized assessment decomposes those mechanisms rather than using group labels as shortcuts.

Social determinants also help explain underdiagnosis. A person can meet symptom criteria yet remain unidentified because care is unavailable, symptoms are normalized within an occupational culture, disclosure threatens employment or legal security, or the available instrument is linguistically or culturally mismatched. Conversely, high service contact can increase the probability that otherwise similar symptoms are documented. Diagnostic datasets therefore contain selection processes: who reaches care, who is screened, who completes assessment, and who receives follow-up. These processes matter for both clinical interpretation and model development. A risk algorithm trained only on treated patients may learn patterns of access rather than population risk, while a prevalence estimate from a displaced or disaster-affected sample may reflect sampling frame and timing as much as underlying burden. Personalized diagnostics should record these pathways explicitly instead of treating missing diagnosis as absence of disorder.

## 5. Integration and Convergence of Genetic and Social Determinants

### 5.1. Gene–Environment Interaction: Sensitivity, Not Destiny

The central convergence model is not “genes plus environment” as two additive lists; it is that environmental exposure can alter the expression of inherited vulnerability, while genotype can modify sensitivity to environmental inputs. *FKBP5* provides the best-developed example: childhood adversity interacts with genetic variation, and trauma-related regulatory changes offer a molecular mechanism through which developmental exposure may have enduring effects [14,15,16]. HPA-axis biology further shows why a snapshot biomarker can be ambiguous. The same cortisol or receptor-related signal may reflect pre-existing vulnerability, recent trauma, chronic adaptation, comorbid depression, medication, sleep disruption, or current social adversity [17].

Differential-susceptibility models extend this reasoning by proposing that some biological characteristics confer greater responsiveness to environments rather than simple vulnerability to harm. For PTSD, this means a variant associated with higher symptoms in severe adversity could, in principle, be neutral or even advantageous in supportive environments. Most available PTSD datasets are not designed to test the full cross-over pattern required for strong differential-susceptibility claims, so the concept should guide hypothesis generation rather than diagnostic labeling. Candidate-gene interaction findings are particularly vulnerable to low power, multiple testing, exposure misclassification, and publication bias; large prospective samples are essential.

### 5.2. Epigenetic Imprinting and the Problem of Causal Direction

Social adversity can become biologically embedded through endocrine, immune, autonomic, metabolic, and epigenetic pathways. PTSD-associated methylation signals in immune-related genes and *AHRR* are compatible with this model, but compatibility is not proof of mediation [34,36,38,39]. Smoking, socioeconomic conditions, cell-type composition, medication, and comorbidity may influence both methylation and PTSD. Cross-sectional case–control designs therefore cannot determine whether an epigenetic difference preceded trauma, resulted from trauma, resulted from PTSD, or reflects a correlated exposure.

Longitudinal designs partially resolve this problem by establishing temporal order. Military cohorts with repeated methylation measures demonstrate the feasibility of detecting trauma-related changes over time [37]. Even then, deployment is a bundle of exposures, and blood is a peripheral tissue. The strongest future studies will integrate pre-trauma baseline, repeated post-trauma phenotyping, exposure timing, cell-resolved or tissue-informed epigenomics, medication and smoking data, and replication in independent populations. Until that standard is common, epigenetic signatures should be described as associated markers rather than mechanistically definitive “trauma scars.”

### 5.3. Biological Embedding of Chronic Social Stress

**Figure 1:**
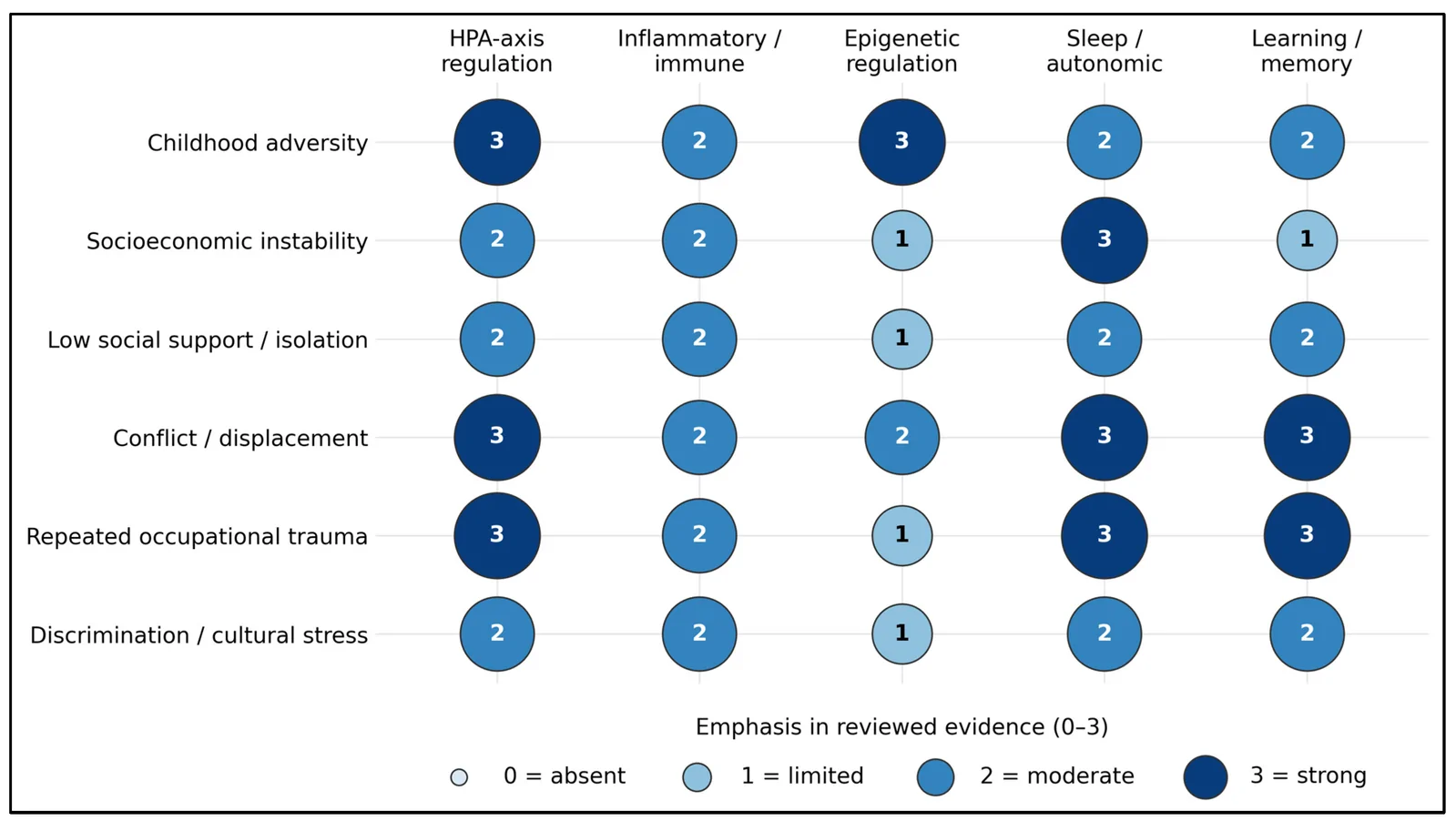
Conceptual map of how social determinants converge on biological embedding pathways relevant to PTSD. Each cell shows the author’s ordinal rating of how strongly the reviewed literature emphasizes that link (1, limited; 2, moderate; 3, strong). Ratings summarize Section 3 and Section 4; they are interpretive rather than pooled effect sizes and identify hypotheses for prospective testing, not established causal pathways.

Chronic social stress can continue after the index trauma through financial insecurity, discrimination, unsafe housing, family conflict, displacement, legal uncertainty, or occupational demands. These conditions may sustain hyperarousal and sleep disruption, alter HPA and inflammatory regulation, and repeatedly reactivate trauma reminders. Social support can buffer some of these processes, whereas isolation can amplify them [46,47]. Thus, the social environment is not merely a distal cause located before diagnosis; it can be part of the mechanism maintaining symptoms and determining whether recovery resources are available. Figure 1 maps these proposed links between social determinants and biological embedding pathways. It summarizes where the reviewed literature places its emphasis and should be read as a set of hypotheses for prospective testing, not as quantitative estimates of effect.

This perspective also changes how “biomarker specificity” should be judged. If a biological signal is responsive to chronic stress across depression, anxiety, infection, obesity, smoking, and PTSD, it may still be prognostically useful, but it is not diagnostically specific. A useful clinical biomarker need not be unique to PTSD if it improves a defined decision such as identifying who requires closer follow-up, but its intended use must be stated explicitly. Conflating susceptibility, state, prognosis, treatment response, and diagnosis is a major reason apparently promising biomarker studies fail to translate.

### 5.4. Intersectionality and Compounded Vulnerability

Intersectionality is clinically relevant when multiple determinants change exposure and access simultaneously. A genetically susceptible individual living in poverty is not simply the sum of a polygenic score and an income variable; poverty can change trauma frequency, nutrition, sleep, neighborhood safety, treatment access, medication adherence, and subsequent stress, while the measured genetic score may itself perform differently by ancestry. Similarly, a refugee may experience premigration violence, family separation, legal insecurity, language barriers, and discrimination after arrival [54,55]. Treating these as independent covariates can hide causal pathways and produce models that are statistically accurate in one dataset yet clinically misleading elsewhere.

Group-level disparities also create a measurement problem. Race/ethnicity, sex, and culture can affect who is exposed, who is recruited into studies, who discloses symptoms, and who receives a clinician diagnosis [48,49,50,57]. A prediction algorithm trained on recorded diagnoses can therefore learn health-system behavior as much as disease biology. Personalized diagnostics will only reduce disparities if the target outcome, sampling frame, and missing-data process are audited rather than treated as neutral.

### 5.5. A Staged Bio-Social Calibration Framework

This review proposes a four-tier framework that separates information by function. Tier 1 characterizes exposure and social context: trauma type, chronicity, developmental timing, ongoing threat, socioeconomic instability, social support, cultural and language context, displacement, and occupational recurrence. Tier 2 establishes the clinical phenotype using validated assessment, ideally clinician-administered CAPS-5 when diagnostic certainty is required and PCL-5 as a screening or monitoring instrument [8,9]. Tier 3 contains biological adjuncts (polygenic scores, methylation, endocrine, inflammatory, and multi-omics measures) that remain research-grade unless validated for a specific population and decision. Tier 4 uses longitudinal symptom and contextual monitoring to detect trajectory change, relapse risk, or delayed expression.

The crucial distinction is calibration rather than criterion modification. A high-risk bio-social profile may justify earlier assessment, more intensive follow-up, repeated screening, or lower thresholds for referral, but it should not lower the symptom threshold required to diagnose PTSD. Likewise, a low-risk profile must not overrule a positive clinical evaluation. This corrects a seductive but unsafe interpretation of “personalized criteria”: adjusting diagnostic criteria to genotype, poverty, race, or social support would embed unvalidated priors into the disease definition and could worsen inequity.

Risk stratification should therefore be evaluated with decision-analytic endpoints rather than association alone. A new variable should demonstrate incremental discrimination and calibration over a strong clinical baseline, reproducibility in independent cohorts, calibration across ancestry and social groups, and a net benefit for a prespecified action. If the intended action is repeated assessment at four weeks, for example, the model should be judged on whether it improves identification of people who later meet full criteria without generating unacceptable false reassurance or unnecessary labeling. The same marker may be useful for one decision and useless for another.

**Figure 2:**
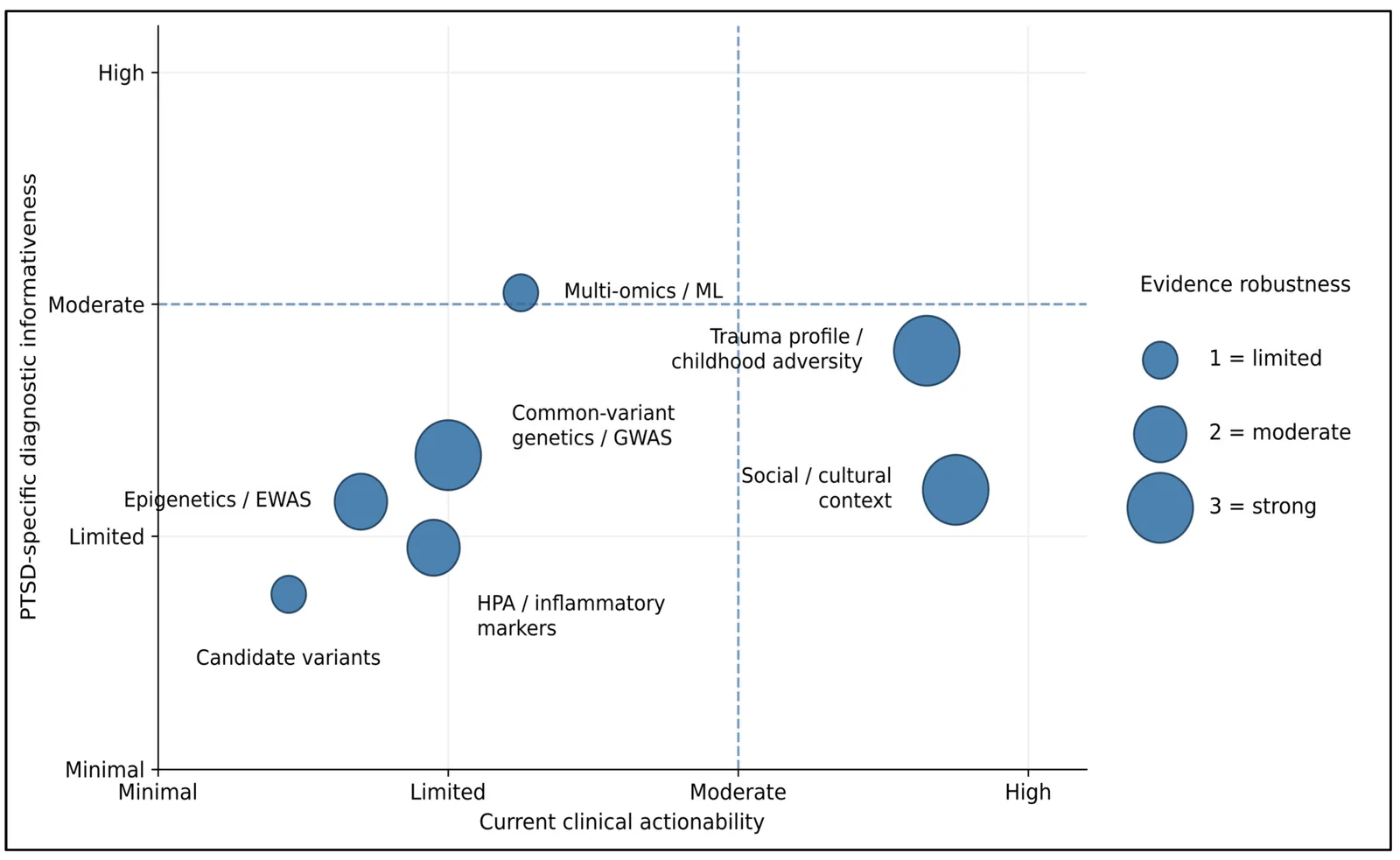
Diagnostic readiness of major PTSD evidence domains. The *x*-axis shows current clinical actionability and the *y*-axis shows PTSD-specific diagnostic informativeness; bubble area reflects the robustness and replication of the evidence base. Positions summarize Table 1 and Section 3, Section 4, Section 5 and Section 6, are interpretive rather than pooled estimates, and are offset slightly within categories to avoid overlap.

Table 1 summarizes the maturity of major evidence domains, while Table 2 translates the framework into a proposed diagnostic workflow. The framework is deliberately conservative: it creates a path for molecular and digital innovation but protects the boundary between research predictors and validated diagnostic evidence. Figure 2 positions each evidence domain by current clinical actionability and PTSD-specific diagnostic informativeness.

## 6. Implications for Personalized Diagnostics

### 6.1. Integrated Risk Scoring and Biomarker-Supported Screening

Biomarker-supported screening is scientifically plausible but clinically premature. Meta-analysis identifies inflammatory differences in PTSD, including higher pro-inflammatory markers at the group level, and prospective work has evaluated C-reactive protein as a risk marker [58,59]. These signals are neither sufficiently specific nor sufficiently stable to diagnose PTSD on their own. Inflammation is altered by infection, obesity, smoking, sleep, medication, physical injury, and many psychiatric conditions; a clinically useful panel would need to demonstrate value beyond these confounders.

Multi-omics studies are closer to the intended precision-diagnostic model because they integrate several weak signals rather than expecting one molecule to carry the diagnosis. A warzone-related PTSD study reported multi-omics biomarker identification and validation, providing an important proof of concept [60]. Translational reviews nevertheless emphasize that diagnostic biomarkers must bridge analytic performance, biological plausibility, and real clinical discrimination [61]. The most recent prospective evidence in children and adolescents remains limited and methodologically heterogeneous, reinforcing that even promising biomarkers require larger representative longitudinal cohorts [62].

### 6.2. Digital Tools, AI, and Early Identification

**Figure 3:**
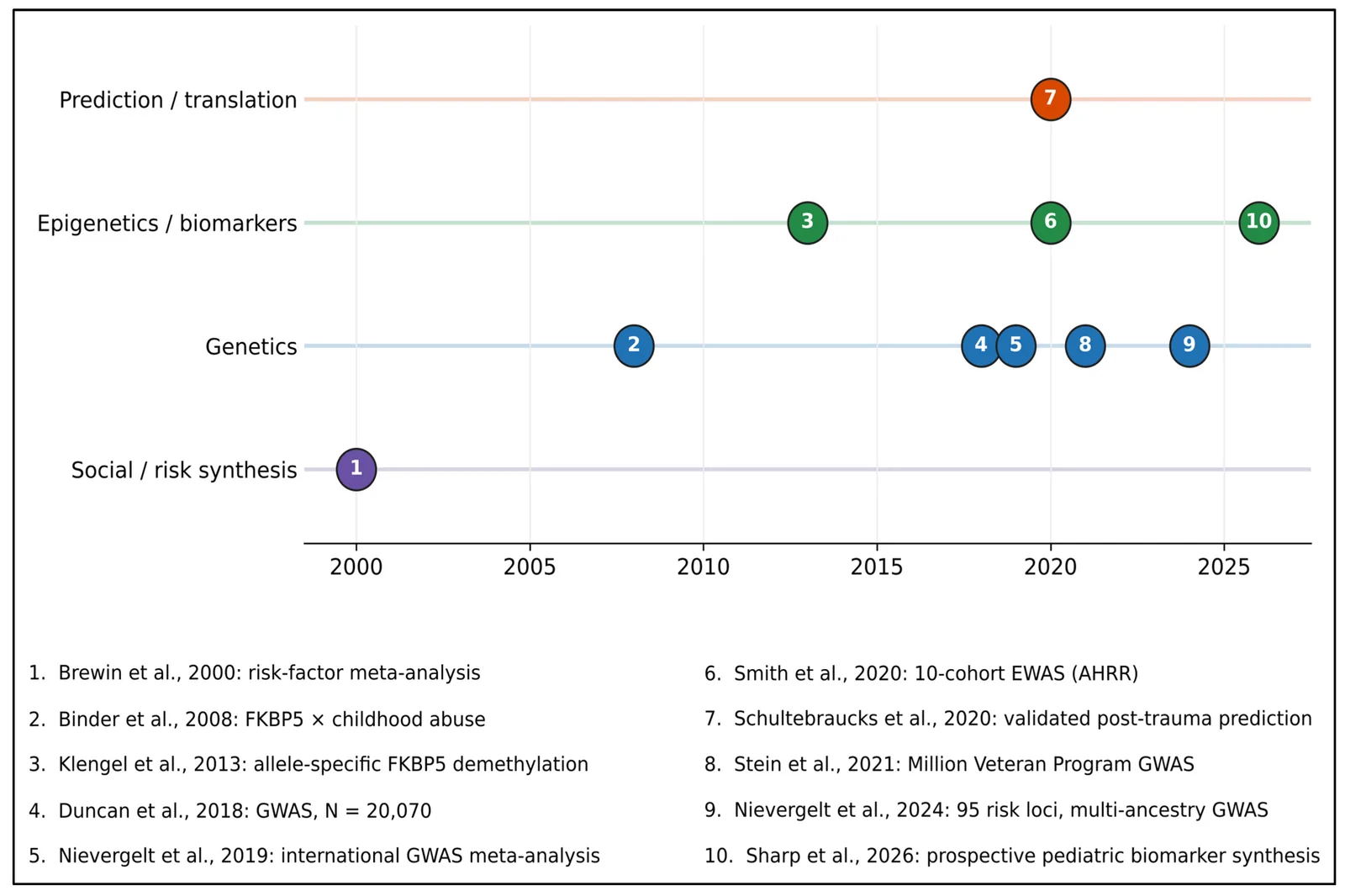
Selected translational milestones in PTSD bio-social diagnostics, from risk-factor synthesis and candidate gene × environment studies to large-scale GWAS, EWAS, and externally validated prediction. Milestones: Brewin et al., 2000 [40]; Binder et al., 2008 [14]; Klengel et al., 2013 [16]; Duncan et al., 2018 [13]; Nievergelt et al., 2019 [28]; Smith et al., 2020 [36]; Schultebraucks et al., 2020 [63]; Stein et al., 2021 [30]; Nievergelt et al., 2024 [32]; Sharp et al., 2026 [62]. AHRR, aryl hydrocarbon receptor repressor; EWAS, epigenome-wide association study; FKBP5, FK506-binding protein 5; GWAS, genome-wide association study.

Machine learning can integrate trauma characteristics, symptoms, demographics, clinical data, and biomarkers without assuming linear effects. A multicohort emergency-department study developed and validated a predictive algorithm for post-traumatic stress course, while earlier work showed that early trauma-response variables could support quantitative forecasting [63,64]. Ensemble models have also predicted later screening status after emergency hospitalization [65]. These studies demonstrate feasibility, not automatic generalizability. Model performance can fall when prevalence, injury mix, measurement schedules, or health-system workflows change. Figure 3 places these prediction studies within the broader translational sequence, from risk-factor synthesis through candidate gene × environment, GWAS, and EWAS milestones. 

**Figure 4:**
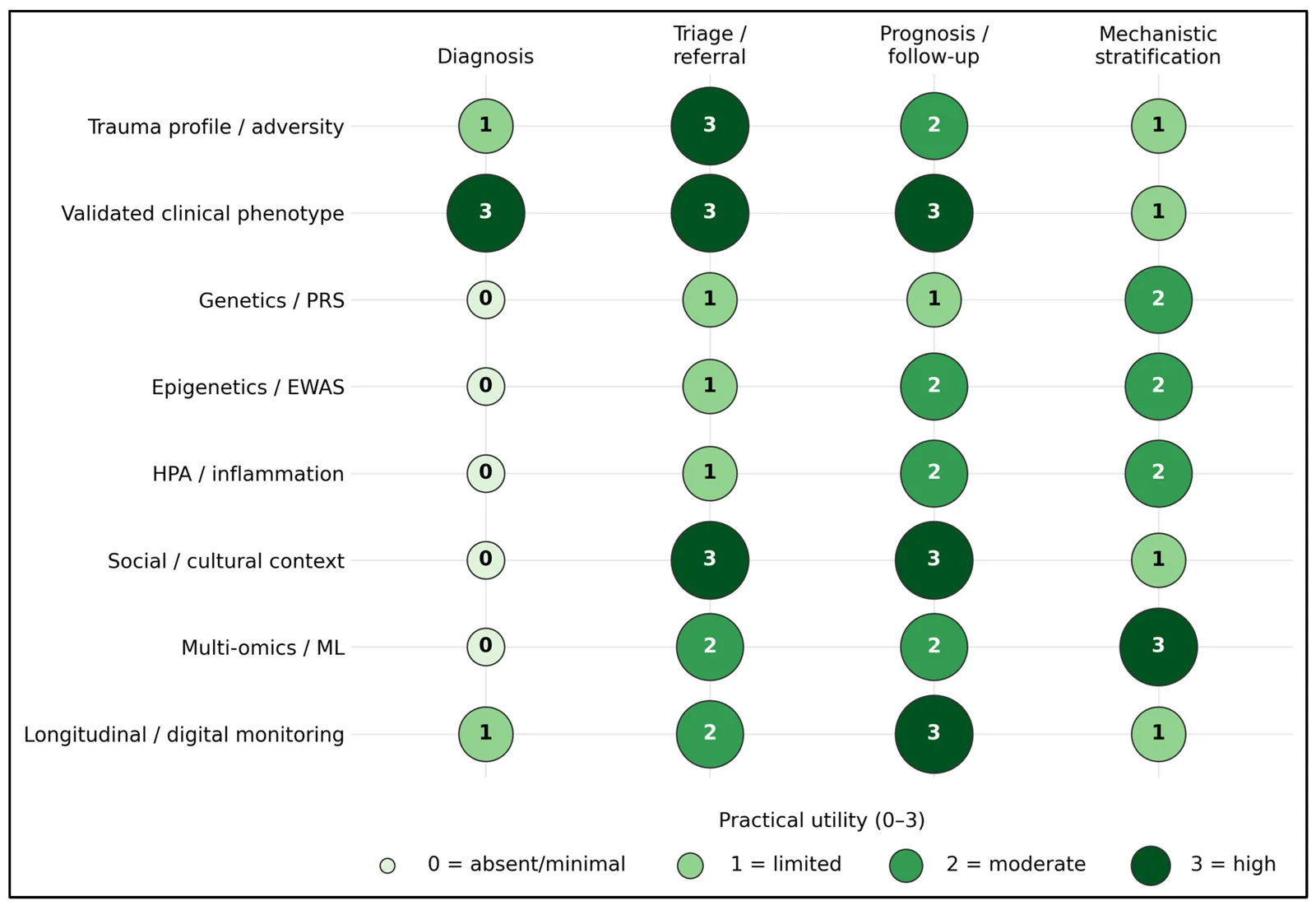
Practical utility of each information source across intended clinical uses. Cell values are the author’s ordinal ratings (0, absent or minimal; 1, limited; 2, moderate; 3, high), synthesizing Section 3, Section 4, Section 5 and Section 6 and Table 1 and Table 2.

The highest-value near-term use is targeted follow-up rather than automated diagnosis: identify recent trauma survivors, military recruits or personnel, displaced populations, abused children, and other high-risk groups who warrant standardized reassessment, while preserving clinician confirmation. Integrated risk scores should report uncertainty and be recalibrated locally. They should also be tested against a simple baseline containing trauma severity, current symptoms, prior psychopathology, and key social determinants; a complex omics model that does not improve decisions over that baseline adds cost without clinical value. Figure 4 summarizes the practical utility of each information source for diagnosis, triage, prognosis, and mechanistic stratification.

### 6.3. Equity, Feasibility, and Ethics

Genetic prediction raises a specific equity problem. Polygenic scores trained predominantly in European-ancestry samples can perform substantially worse in other ancestries, and uncritical clinical use can exacerbate health disparities [66]. Diverse-ancestry GWAS require careful methods for population structure, linkage disequilibrium, allele-frequency differences, and interpretation [67]. A PTSD algorithm that combines ancestry-sensitive genetic scores with socially patterned clinical data could therefore compound two sources of bias.

Algorithmic fairness cannot be solved by removing race from the input. Health-system data encode prior access and resource allocation; a widely cited clinical example showed that an algorithm using cost as a proxy for need reproduced racial inequity [68]. PTSD systems face analogous risks if recorded diagnosis, health-care utilization, employment status, or treatment uptake are treated as unbiased labels. Consent for genetic data, privacy, stigmatization, potential occupational misuse, data security, assay cost, laboratory access, language coverage, and clinician interpretability must be evaluated alongside accuracy.

Accordingly, “tailored diagnostic pathways” should mean tailoring the pathway around the criteria, not tailoring the criteria themselves. Bio-social profiles can determine who is screened, how soon they are reassessed, whether interpreters or culturally adapted instruments are needed, and what practical barriers must be addressed. Multi-omics and population-level screening should remain research or implementation-science objectives until prospective studies show transportable calibration, clinical utility, and equitable net benefit.

**Figure 5:**
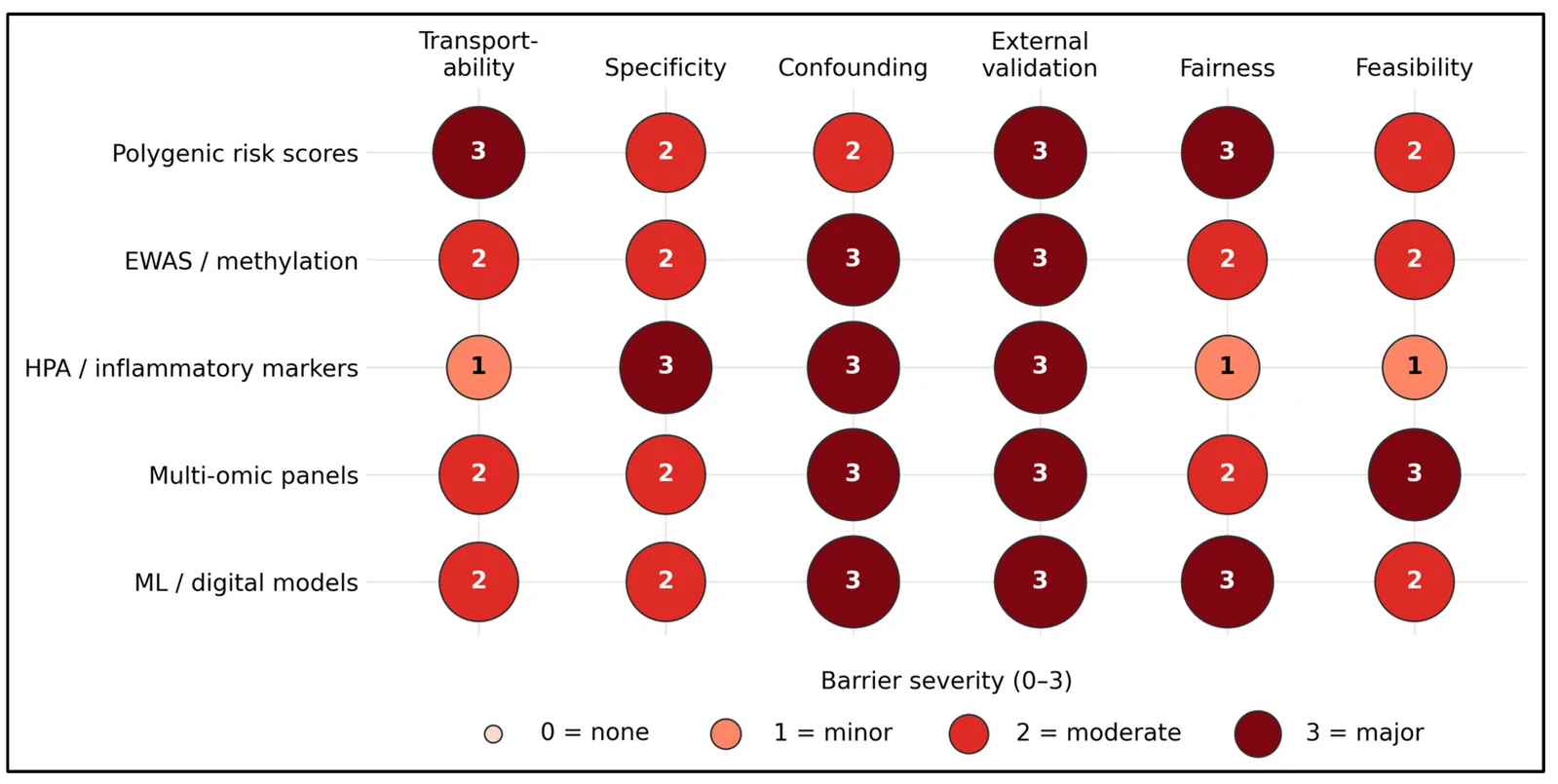
Implementation barriers for candidate precision-diagnostic modalities. Cell values are the author’s ordinal ratings of barrier severity (1, minor; 2, moderate; 3, major) across transportability, specificity, confounding, external validation, fairness, and feasibility.

Clinical feasibility should be judged against the decision the tool is meant to improve. Low-cost social and symptom variables can be collected immediately and repeatedly; genomic and multi-omics assays require laboratory infrastructure, quality control, data governance, and interpretable reporting. A staged implementation strategy is therefore more defensible than an all-at-once precision panel: first standardize trauma and symptom phenotyping, then test whether biological data add enough value to justify their cost and complexity. External validation should precede deployment, and post-deployment monitoring should examine calibration drift, subgroup error rates, referral consequences, and whether the system actually reduces missed cases without creating excessive false positives. Figure 5 profiles the principal translational barriers for each candidate modality.

### 6.4. Limitations

This review has limitations. It is a targeted integrative review rather than a registered systematic review, and screening, selection, and appraisal were performed by a single reviewer, so selection bias cannot be excluded. The search was restricted to English-language studies, and the evidence base is dominated by cohorts from the United States and Europe, which limits transportability to other settings. The ordinal ratings in Figure 1, Figure 2, Figure 4 and Figure 5 are single-rater interpretive judgments; independent rating by a second reviewer, with agreement statistics, would strengthen future syntheses. Finally, no formal risk-of-bias tool was applied, although each source was appraised for design, sample size, replication, and external validation.

## 7. Conclusions

PTSD emerges from the convergence of trauma exposure, inherited liability, developmental history, biological adaptation, and the social conditions in which threat and recovery occur. Genetic research has progressed from fragile candidate-gene claims toward large polygenic GWAS, and epigenetic studies are moving toward larger, longitudinal, multi-cohort designs. Social evidence is already clinically consequential: trauma chronicity, childhood adversity, support, socioeconomic instability, culture, displacement, and occupational context shape risk, persistence, disclosure, and access to care. None of these determinants, alone or in combination, currently warrants replacing a standardized clinical diagnosis.

The main implication for personalized diagnostics is therefore one of architecture. The clinical phenotype should remain the diagnostic anchor, while bio-social information calibrates who is assessed, when assessment is repeated, how uncertainty is interpreted, and what barriers or continuing exposures must be addressed. Genetic, epigenetic, endocrine, inflammatory, and multi-omics markers belong in an evidence-staged adjunct layer until prospective studies demonstrate incremental utility over strong clinical baselines. A high-risk profile should increase vigilance; a low-risk profile should never negate clinically established PTSD.

The most important research gap is not the absence of additional associations. It is the shortage of longitudinal, multi-ancestry, socially diverse cohorts that measure exposure before and after trauma, harmonize PTSD phenotyping, collect multi-omics data at biologically meaningful times, and test models in independent health systems. Future studies should prespecify intended use, calibration, subgroup performance, net clinical benefit, and consequences of false-positive and false-negative decisions.

A precision-based PTSD assessment system is achievable only if precision means better-calibrated clinical decisions rather than molecularized diagnosis. The proposed bio-social calibration framework provides a defensible route: preserve validated diagnostic criteria, integrate social context as actionable clinical information, evaluate biological markers according to readiness, and require transportability and fairness before scale-up. That approach offers a realistic opportunity to improve early identification and reduce rather than encode diagnostic disparities.

## Figures and Tables

**Table 1:** Evidence domains and current diagnostic readiness in PTSD.

Domain	Representative Evidence	Current Diagnostic Maturity	Major Limitation
Common-variant genetics/GWAS	Large consortia support polygenic architecture and many risk loci.	Etiologic and stratification research; not a stand-alone diagnostic test.	Small distributed effects; phenotype heterogeneity; ancestry portability.
Candidate variants (e.g., FKBP5, 5-HTTLPR, COMT, BDNF)	Biologically plausible associations and some meta-analytic signals.	Research only.	Replication, exposure interaction, multiple testing, small effect size.
Epigenetics/EWAS	Multi-cohort methylation signals, including immune-related loci.	Research only; potentially state/trajectory informative.	Tissue specificity, smoking/cell composition, reverse causation.
HPA-axis and inflammatory markers	Group-level endocrine and inflammatory differences reported.	Adjunctive research biomarkers.	Low disorder specificity; timing, medication and comorbidity effects.
Trauma profile and childhood adversity	Consistent epidemiologic risk gradients.	Clinically actionable context and triage information.	Not diagnostic; exposure measurement and recall bias.
Social support, socioeconomic and cultural context	Strong associations with persistence, help-seeking and recovery resources.	Clinical formulation and follow-up planning.	Confounding, bidirectionality, setting-specific measurement.
Multi-omics/machine learning	Proof-of-concept classification and prediction in selected cohorts.	Promising but requires external prospective validation.	Overfitting, dataset shift, missingness, fairness and implementation.

**Table 2:** Proposed bio-social calibration framework for personalized PTSD assessment.

Tier	Information	Intended Use	Current Status/Safeguard
1. Exposure and social context	Trauma type/dose; ongoing threat; childhood adversity; socioeconomic instability; support; culture/language; displacement; occupation.	Set pretest risk; identify barriers; plan timing and intensity of follow-up.	Use clinically now, but never treat contextual risk as diagnostic evidence.
2. Validated clinical phenotype	CAPS-5 for diagnostic assessment; PCL-5 for screening/monitoring; impairment and differential diagnosis.	Establish or exclude current PTSD; quantify symptom burden.	Clinical core. Standardize administration and use validated translations.
3. Biological adjuncts	PRS, EWAS/methylation, cortisol/HPA markers, inflammation, multi-omics panels.	Research stratification; test incremental value and mechanisms.	Do not override clinical criteria; require analytical validity, external validation and ancestry portability.
4. Longitudinal/digital monitoring	Repeated symptoms, exposure changes, functioning, treatment uptake; validated digital signals where available.	Detect delayed trajectory, relapse, non-recovery and changing care needs.	Use transparent consent, missing-data audits, recalibration and human review.

## Data Availability

No new datasets were generated or analyzed for this review.
